# Focused ion and electron beams for synthesis and characterization of nanomaterials

**DOI:** 10.3762/bjnano.16.47

**Published:** 2025-05-02

**Authors:** Aleksandra Szkudlarek

**Affiliations:** 1 Academic Centre for Materials and Nanotechnology, AGH University of Krakow, av. Mickiewicza 30, 30-059, Krakow, Poland

**Keywords:** deposition, etching, focused electron beams, focused ion beams, lithography, milling, nanofabrication, 3D nanostructures

It is challenging to discuss the fabrication of small devices without acknowledging Richard Feynman, the pioneer of nanotechnology, and his iconic lecture, "There’s Plenty of Room at the Bottom" [1], delivered at the annual meeting of the American Physical Society at Caltech in 1959. The central theme of his talk revolved around manipulating and controlling matter at an extremely small scale to address a crucial technological question: "Why can't we manufacture small devices?" [1]. According to Feynman, there were no unbeatable obstacles from a scientific perspective; the limitation laid in the underdeveloped technology at that time. In his visionary address, he proposed techniques such as direct ion lithography using focused beams and introduced the concept of combined photo-electron lithography.

Feynman's contribution was not limited to his visionary approach of advancing technology through scientific discovery; he also actively encouraged innovation by offering a $1,000 prize to anyone who could scale down a page of text from a book by a factor of 25,000. The prize remained unclaimed for 25 years until Tom Newman succeeded in etching the opening lines of Dickens’ A Tale of Two Cities *–* "It was the best of times, it was the worst of times" [2] – onto a 200 × 200 micron square of plastic using an electron beam. This achievement prompts a reflection: is nowadays the best or the worst of times for the development of electron and ion beam technologies?

With the hope to contribute addressing this question, the co-guest editors Dr. Ivo Utke, Dr. Katja Höflich, Dr. Gregor Hlawacek, Dr. Nico Klingner, and myself organized a thematic issue in connection with the work presented at the joint meeting of the FIT4NANO (fit4nano – Focused Ion Technology for Nanomaterials) and FEBIP (Focused Electron Beam Induced Processing) communities during the Focused Charged Particle Week held in Krakow in 2022.

Over the past decade, following the publication of the comprehensive book edited by Ivo Utke, Stanislav Moshkalev, and Philip Russell on the fundamentals and applications of focused electron and ion beams [3], charged particle beams have been instrumental in advancing modern science and technology. These beams are essential for both fabricating and characterizing materials, enabling researchers to probe fundamental properties of matter, investigate atomic-scale processes, and design novel materials with tailored 3D architectures [4].

Focused beams not only allow the characterization of atomic structures but also enable precise local modification of material properties through ion milling and the creation of novel structures with tunable mechanical, electrical, and magnetic properties using gas-assisted etching or deposition. The precision and versatility of these beams, including the use of multiple gas species, open pathways to fabricate 3D nanomaterials that are unattainable through conventional chemical methods. However, achieving reproducibility in such structures requires a deep understanding of the fundamental mechanisms underlying precursor fragmentation by low-energy electrons, which remains an ongoing focus of study in the field, see Figure 1.

**Figure 1:**
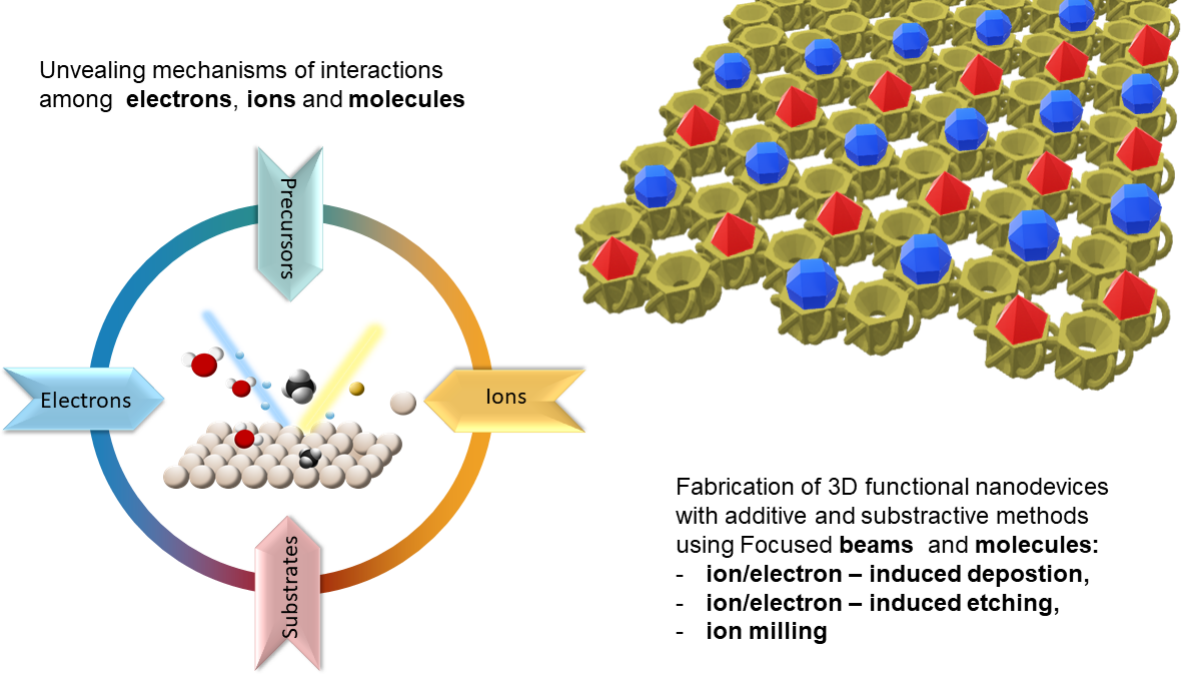
Complexity of interactions among electrons, ions, and precursor molecules behind fabrication of functional 3D nanostructures.

Within the widely studied group of acetylacetonate complexes, which also play a crucial role in chemical vapor deposition and atomic layer deposition techniques, this thematic issue includes studies on low-energy electron interactions with metal(II) bis(acetylacetonate) complexes [5].

Another molecule investigated for its gas-phase fragmentation mechanism via dissociative ionization and dissociative electron attachment is [Au(CH_3_)_2_Cl]_2_. Studies by Oddur Ingólfsson’s group [6], supported by quantum chemical calculations, revealed that chlorine removal during focused electron beam induced deposition (FEBID) was nearly complete, in contrast to the limited chlorine loss observed in gas-phase experiments. Previous studies have shown that gas-phase investigations of molecular fragmentation by electron beams may not fully predict the behavior of molecules when adsorbed on a surface. This highlights the importance of comparing gas-phase fragmentation results with FEBIP processes. In this issue, the novel iron precursor (i.e., iron tetracarbonyl methyl acrylate (Fe(CO)_4_MA)) is studied in the gas phase using mass spectrometry and during electron-induced processes [7–8].

This issue also presents insights from initial studies using the novel (hfac)AgPMe_3_ precursor [9], which notably demonstrates a high level of purity at the interface with the substrate. Metallic structures can be fabricated directly or through post-purification methods, such as water-assisted treatment, which has been effective for Au and Pt deposits [10]. Interestingly, morphological changes in the underlying SiO_2_ layer were observed during the process, resembling effects noted in water-assisted graphene etching [11].

While controlling the purity of deposits appears more straightforward, achieving precise nanoscale shapes remains challenging, particularly when structures are fabricated in close proximity. Experimental results demonstrate that FEBID, followed by focused electron beam-induced etching (FEBIE), can effectively control edge profiles, supported by continuum modeling [12]. Additionally, using alternative precursors such as Pt(CO)_2_Cl_2_ and Pt(CO)_2_Br_2_ with low-energy ion irradiation enables the fabrication of high-purity Pt deposits. This process involves CO desorption and halogen removal, overcoming the limitations of standard MeCpPtMe_3_ precursors, which yield low metal content [13].

In contrast to electrons, ions interact more energetically, generating local high temperatures that can damage sensitive samples such as biological or polymeric materials. This damage can be mitigated by using low energy focused ion beams, which allow for higher currents, faster processing speeds, and reduced sample degradation [14]. This thematic issue also includes studies on soft DNA origami nanostructures, showcasing methods to preserve their integrity during ion beam interactions [15]. In addition, the extension of the continuum model has proven useful in predicting the outcomes of ion beam milling processes in multilayered systems, as demonstrated in the case of the Si/SiO_2_/Pt system [16]. To identify the current state of the technology and routes towards its further development, a comprehensive roadmap for focused ion beam technologies has been recently published by an interdisciplinary group of international experts. Although not part of this thematic issue, we encourage the reader to take a look at the original paper [17]. While achieving unrivaled spatial resolution and 3D capabilities, focused ion and electron beams technologies face challenges in reproducibility and scalability, hindering their commercial applications. Hence, these techniques require better understanding of the atomistic mechanisms involving ions, electrons, adsorbates, and substrates to transform them from specialized prototyping to market-relevant methods. Advances are particularly needed in precursor development to achieve desirable material compositions and in modeling to enable full 3D-growth control. As we transition into an era defined by artificial intelligence and automation, the potential for real-time process control and modeling-informed precision fabrication is immense. Rapid data analysis and automated software development could pave the way for a bottom-up approach, akin to how nature constructs complex biological systems.

On behalf of all editors,

Aleksandra Szkudlarek

Krakow, April 2025

## Data Availability

Data sharing is not applicable as no new data was generated or analyzed in this study.
